# Addressing Weight‐Related Bullying in Schools: Youth Perspectives on School Policies and Interventions

**DOI:** 10.1111/josh.70054

**Published:** 2025-08-29

**Authors:** Ritika Kale, Erin N. Harrop, Jaylyn R. Kelly, Sarah A. Sullivan, Kendrin R. Sonneville

**Affiliations:** ^1^ Lyman Briggs College, Michigan State University East Lansing Michigan USA; ^2^ Graduate School of Social Work, University of Denver Denver Colorado USA; ^3^ Department of Family Medicine, University of Michigan Ann Arbor Michigan USA; ^4^ Department of Nutritional Sciences, University of Michigan School of Public Health Ann Arbor Michigan USA

**Keywords:** anti‐bullying policy, school bullying, weight bullying, weight stigma

## Abstract

**Background:**

Weight‐related bullying is linked to negative mental health outcomes. However, anti‐bullying policies targeting weight‐based bullying remain limited. This study aimed to gather youth perspectives on weight‐related bullying and potential school interventions.

**Methods:**

Data were collected in November 2022 from the MyVoice National Poll of Youth, a diverse cohort of U.S. youth (ages 14–24). Respondents (*n* = 621) answered five open‐ended questions about weight‐related bullying, which were analyzed using inductive content analysis.

**Results:**

A majority of respondents (71.6%) reported witnessing weight‐related bullying. While most respondents attributed bullying to characteristics of the individual doing the bullying (e.g., insecurity), characteristics of the target of the bullying were more commonly named for weight‐related bullying compared to bullying in general (46.5% vs. 29.2%; *p* = 0.0013). Respondents suggested both educational/programming approaches and policy solutions. Most respondents (68.5%) supported the inclusion of weight‐related bullying in school anti‐bullying policies.

**Implications for School Health Policy, Practice, and Equity:**

Research on anti‐bullying laws shows potential benefits for youth (e.g., reduced bullying, improved mental health). As policies addressing weight‐related bullying expand, research is needed to assess their effectiveness and minimize unintended consequences.

**Conclusions:**

Youth support the inclusion of weight‐related bullying in school policies, which could help create more supportive environments and improve mental health outcomes.

Bullying is a pervasive issue among youth and a significant public health concern, linked to negative outcomes in education, psychological well‐being, physical health, and social relationships, with long‐lasting effects into adulthood [1]. Approximately 20% of youth experience bullying at school, most commonly through spreading rumors or being made fun of or called names. Bullying often occurs in person within school settings (e.g., classrooms, hallways, cafeterias, or outdoor spaces), but may also take place online or via text during school hours. When bullying is related to personal characteristics, appearance is the most frequently cited reason [2]. A meta‐analysis of over 113,000 youth found that larger‐bodied youth are at a higher risk of being bullied compared to their thinner peers [3]. Research suggests that weight is the most common reason youth are bullied [3], with more than one‐third of adolescents reporting experiencing weight‐related teasing [4]. This issue disproportionately affects higher‐weight youth, Black, Indigenous, and People of Color (BIPOC) youth, and those from low socioeconomic backgrounds [4].

Higher weight youth are more likely to experience negative mental health outcomes, including suicidality, depression, and eating disorders [5, 6]. However, research suggests that it is not the child's weight itself that leads to these poor mental health outcomes. Rather, it is the child's body image and experiences with weight‐related bullying and discrimination that are more strongly associated with these negative mental health outcomes [6, 7, 8]. As an example, higher weight children with positive body image who do not experience bullying do not exhibit these same adverse outcomes [7]. Adolescents who are teased for their weight are at an increased risk for behaviors such as chronic dieting, engaging in extreme weight control practices, and binge eating [4]. Moreover, adolescents who face body‐related stigma are 2.5 times more likely to experience thoughts of self‐harm and twice as likely to engage in self‐harm, contemplate suicide, and attempt suicide [8].

Legal efforts have shown promise in addressing bullying and its associated mental health impacts. For instance, a 2022 study found that state‐level anti‐bullying laws reduced bullying, as well as depression and suicidal ideation among adolescents, with marginalized youth (e.g., girls, queer students) experiencing greater benefits [9]. While every U.S. state has an anti‐bullying law, the comprehensiveness of school policies varies widely and the student identities explicitly protected (e.g., disability, race, gender, sexual orientation) differ across states. Only four states have policies that include explicit protections against appearance‐ or weight‐related bullying [10, 11], despite the disproportionately high rates of bullying among larger youth and the severe, long‐lasting consequences they face [3, 4, 8, 12]. These gaps in policy make it harder for schools to effectively address weight‐related bullying. Enumeration of specific student identities in anti‐bullying legislation has been shown to strengthen schools' ability to set pro‐social norms and combat peer abuse [11]. Highlighting the value of enumeration, one recent study found that educators in school districts with anti‐bullying policies that specifically addressed weight demonstrated lower levels of weight bias than educators in districts without weight‐enumerated policies [13].

Despite limited policy action addressing weight‐related bullying, research shows that school personnel are generally supportive of these policies [14, 15]. For example, one study found that 92% of educators supported anti‐bullying policies that specifically target weight‐related bullying [15]. Additionally, studies of youth suggest a strong need for more effective interventions [14, 16]. In one study, most students who had been the target of weight‐related bullying expressed a desire for support from others, including parents, peers, and teachers [16]. However, no previous studies have explicitly examined youth perspectives on policy‐driven solutions to weight‐related teasing. This study seeks to fill this gap by exploring youth perspectives on the frequency, causes, and prevention strategies for weight‐related bullying.

## 1 Methods

### 1.1 Participants

Data for this study came from the MyVoice National Poll of Youth, a large cohort of young people (ages 14–24 years) across the United States [17]. Participants were recruited through in‐person engagement at community and youth‐oriented events, as well as through targeted advertisements on Facebook and Instagram, aiming to recruit a diverse sample reflective of U.S. demographics based on American Community Survey benchmarks. Demographic data, including self‐reported age, gender, race/ethnicity, parental education level, and eligibility for free/reduced lunch, were collected during study enrollment. Participants provided online consent or assent, with a waiver of parental consent for minors, under approval from the [redacted] Institutional Review Board.

### 1.2 Instrumentation

This study utilized responses to a set of questions about weight‐related bullying that were sent to 796 MyVoice participants in November 2022. The set included five open‐ended questions, which were sent via SMS text message to individual MyVoice participants:

What do you think are common reasons students bully or tease others?

Have you ever seen someone being bullied or teased because of their weight? Tell us about it.

Why do you think students bully or tease others about their weight?

What do you think schools can do to stop weight‐related bullying and teasing?

Do you think anti‐bullying policies at schools should specifically include weight‐related bullying and teasing? Why or why not?

### 1.3 Procedure

MyVoice participants receive weekly surveys on health‐related topics relevant to youth and are compensated $1 for each completed survey. Survey questions are developed and refined collaboratively by the MyVoice team, which includes experts in adolescent health, family medicine, health behavior, nutrition, qualitative research, and mixed‐methods research, as well as high school, undergraduate, and graduate‐level trainees. The development process prioritizes youth perspectives, ensuring relevance and appropriate tone for the MyVoice audience. Each set of questions undergoes a pilot phase, during which they are tested via text message with current and former team members for final review and feedback prior to implementation. While other question formats may be used, MyVoice surveys primarily rely on open‐ended questions to support a conversational tone and better align with the SMS platform. Forced‐choice response options can be confusing in text‐based formats and are generally less effective for capturing the richness of youth perspectives. Each survey topic, including the questions and their wording, can be accessed from the MyVoice online question bank (hearmyvoicenow.org/questionbank/). Questions are administered weekly via SMS text message in English, with responses securely collected via an online platform. Responses are then downloaded as CSV files and de‐identified, with all personally identifiable information (including names and locations) removed prior to analysis.

### 1.4 Data Analysis

Inductive content analysis was used to analyze the responses [18, 19]. After cleaning and de‐identifying the data, three analysts (RK, JK, KRS) independently reviewed all responses to all questions and drafted preliminary lists of common ideas captured in responses (i.e., memoing). Ideas were refined through a comprehensive review and discussion among the team members to ensure clarity and consensus. The team then developed detailed narrative descriptions of the most commonly endorsed ideas and actionable insights for each question, along with illustrative quotes to support consistent coding (see the Results section for a description of codes by question). Divergent perspectives were captured through negative case analysis to add nuance to the overarching narrative. To enhance consistency in the coding process, the analysts independently coded a shared set of 10 responses and compared their findings. Discrepancies in the coding were discussed, and adjustments were made to the codebook to enhance its clarity and consistency. Following this process, a final version of the codebook was applied to the entire dataset. Five coders (RK, KC, CD, GW, SK) independently coded the complete dataset, with each response being coded by two separate analysts. Any disagreements between coders were resolved through external review by a doctoral‐level expert (SAS) with expertise in weight‐related bullying.

## 2 Results

Of the 796 MyVoice participants who received the question set on weight‐related bullying, 621 respondents (78%) provided a codable response to at least one question. Uncodable responses were rare and included undecipherable strings of letters or symbols or responses that could not be interpreted as a response to the proposed question.

Demographic characteristics of the respondents are presented in Table 1. In response to the question about past exposure to weight‐related bullying (Q2: “Have you ever seen someone being bullied or teased because of their weight? Tell us about it.”), 71.6% of respondents indicated they had witnessed someone being bullied or teased because of their weight. Of these respondents, 28.9% shared specific examples of in‐person, non‐school bullying, 20.0% provided examples of school‐based bullying, and 7.7% described instances of online bullying.

**TABLE 1:** Demographic characteristics of respondents (*n* = 621).

Demographic characteristic	Category	*N* (%)
Gender	Male	256 (41.2)
	Female	300 (48.3)
	Non‐binary, transgender, or other identity	65 (10.5)
Age	14–17	106 (17.1)
	18–21	352 (56.7)
	22–24	163 (26.2)
Race/ethnicity	Mixed race, non‐hispanic	40 (6.4)
	Black or african american, non‐hispanic	60 (9.7)
	White or caucasian, non‐hispanic	343 (55.2)
	Asian, non‐hispanic	99 (15.9)
	American indian/native hawaiian, non‐hispanic	2 (0.3)
	Hispanic/latino, all races	77 (12.4)
Parental education	Less than bachelor's degree	218 (35.1)
	Bachelor's degree or above	401 (64.6)
	Missing	2 (0.3)
Qualified for free or reduced lunch	Yes	226 (36.4)
	No	390 (62.8)
	Missing	5 (0.8)

### 2.1 Perceived Reasons for Bullying

Perceived reasons for bullying were explored through two questions: one about general bullying (Q1: “What do you think are common reasons students bully or tease others?”) and one focusing on weight‐related bullying (Q3: “Why do you think students bully or tease others about their weight?”). An identical coding scheme was applied to both questions, as responses revealed similar patterns (see Table 2 for code descriptions, illustrative quotes, and frequencies). Illustrative quotes included in Table 2 and in the text were drawn from responses to one of the two questions, but were selected because they exemplify responses observed across both questions.

**TABLE 2:** Reasons for bullying^a^ response codes.

			Frequency, *N* (%)	
Code title	Description	Illustrative quote	General bullying (*n* = 621)	Weight‐related bullying (*n* = 565)
Characteristics of the individual doing the bullying; Bullying motivation	This code captures traits and motivations of individuals who engage in bullying. Characteristics may include personal insecurities, low self‐esteem, jealousy, anger, perceived superiority, or struggles such as mental health issues, learning disabilities, or a lack of emotional intelligence or empathy. Motivations for bullying might include a desire to gain social status, attention, power, or dominance, or actions driven by boredom, fun, or to boost their own self‐esteem at the expense of others.	*“Insecurity, fear of alienation, and lack of emotional intelligence/coping mechanisms”*	446 (71.8%)	317 (56.0%)
Characteristics of the target of the bullying; Societal standards	This code captures perceptions of traits that may make individuals more vulnerable to bullying, based on societal standards or norms. It includes factors such as non‐conformity to prevailing ideals related to appearance, behavior, or identity (e.g., fatphobia, racism, sexism, homophobia, transphobia), as well as being perceived as “different” or an “easy target.” It may also reflect attitudes that blame the target of the bullying, suggesting they “deserve” the bullying due to their differences	*“I think students bully or tease others for appearing or behaving in a way different from societal norms”*	178 (28.7%)	263 (46.5%)
Family life/Upbringing	This code captures how external factors such as family dynamics, home life, and parental or role model influences may contribute to bullying behavior. It includes factors such as exposure to conflict, neglect, abuse, or unhealthy family relationships, which might shape a person's attitudes and actions.	*“Because they have a bad family life”*	124 (20.0%)	55 (9.7%)
Past bullying	This code captures how previous experiences of being bullied may influence individuals to engage in bullying behavior themselves.	*“Cause they were bullied themselves”*	34 (5.5%)	5 (0.9%)
Media/Social media	This code captures the influence of both social media and traditional media on bullying behaviors. It includes direct mentions of bullying occurring on online platforms, as well as how media portrayals or content may shape attitudes toward bullying.	*“Standards set by social media”*	5 (0.8%)	10 (1.8%)
Uncategorized	This code includes responses where participants express uncertainty or provide answers that do not clearly align with any of the codes.	*“I'm not sure”*	11 (1.8%)	28 (4.9%)

^a^ Codes generated from a content analysis of two questions pertaining to reasons for general bullying (Q1: “What do you think are common reasons students bully or tease others?”) and weight‐related bullying (Q3: “Why do you think students bully or tease others about their weight?”). Responses may receive multiple codes, so column totals are > 100%.

The majority of participants attributed bullying to characteristics of the individuals doing the bullying or their motivations. Responses focused on characteristics of the individual doing the bullying accounted for 71.8% of general bullying responses and 56.0% of weight‐related bullying responses. These responses highlighted traits such as insecurity, low self‐esteem, jealousy, perceived superiority, or mental health struggles (e.g., “Because they are jealous and insecure about themselves, and because they aren't used to people different than them”*)*. Motivations of individuals doing the bullying were often described in terms of the desire to fit in, gain attention, or for entertainment (e.g., “I think to fit in with others, because they have a low self‐worth and need to take that out on others, and because they can”*)*.

A significant portion of respondents also linked bullying to characteristics of the target of the bullying. Target‐focused responses made up 28.7% of general bullying responses and 46.5% of weight‐related bullying responses. Among the 559 respondents who answered both questions, target‐focused responses were significantly more commonly given as a reason for weight‐related bullying compared to general bullying (46.5% vs. 29.2%; *p* = 0.0013). These responses suggested that bullying often targeted individuals who were “different,” did not conform to societal norms, or were seen as “easy targets.” Some responses explicitly blamed the target of the bullying (e.g., “Because they deserve it”). Less frequent explanations for bullying included influences from family (e.g., “Insecurities and prejudice from their families”), past experiences of being bullied (e.g., “They've been bullied themselves”), and the impact of media/social media (e.g., “I think it's because of social media tends to display people who fit beauty standards and thus students pick on those who don't fit in”).

### 2.2 Strategies to Prevent School‐Based Bullying

Responses to one question (Q4: “What do you think schools can do to stop weight‐related bullying and teasing?”) were used to generate ideas for preventing school‐based bullying (see Table 3 for code descriptions, illustrative quotes, and frequencies). The two most common strategies identified were addressing education/programming at schools (44.9%; e.g., “I think schools can implement programs to help students deal with body image issues and disordered eating.”) and policies/consequences (30.8%; e.g., “Not just giving warnings for verbal harassment and bullying, but immediate consequences”), with some respondents suggesting both approaches (e.g., “Encourage health education regarding weight and associated factors, address and punish behaviors that encourage or include weight‐related bullying/teasing”). Other suggestions included discontinuing weight‐focused practices (7.3%; e.g., *“There are some programs they could stop doing, like anything involving weighing students, especially in front of each other. having the nurse do weights and sending home letters for those with a high bmi*.*”*), increasing support services for targets of bullying (6.4%; e.g., “Do mental check ins with the students that are bullied and teased”), and role modeling within the school setting (4.5%; e.g., “Schools can show more diverse representation”). Despite these suggestions, 16.5% of respondents believed it was unrealistic for schools to stop weight‐related bullying and framed the issue as requiring systemic or societal change (e.g., “I think it is difficult for schools to stop it, it needs to be a societal change. Everything that we are shown reflects a preference towards being skinny.”). An additional 5.2% of responses included ideas that blamed the target of the bullying, either by insinuating that their “bad” habits are to blame (e.g., “maybe force kids to eat actual healthy food rather than pizza and chicken nuggets every day”) or by suggesting weight loss as a solution to weight‐related bullying (e.g., “Offer resources for weight loss”).

**TABLE 3:** Strategies to prevent weight‐based bullying^a^ response codes.

Code title	Description	Illustrative quote	Frequency, *N* (%) (*n* = 559)
Education/programming	This code refers to educational initiatives aimed at addressing weight‐related bullying in schools. It includes health education (e.g., nutrition, mental health), programs that promote inclusivity, kindness, self‐acceptance, body positivity, and body diversity, as well as curriculum changes that focus on teaching these values. It also encompasses recommendations for bystander training or bias education to help prevent bullying and encourage supportive behaviors among students.	*“Education that instills the idea that people of all weights are not superior or inferior to one another bc of their weight”*	251 (44.9%)
Policies/consequences	This code refers to school policies and actions related to bullying prevention and response. It includes discussions of disciplinary measures, such as punishment or other consequences for bullying, both in the moment and after the fact. It also captures recommendations for policies like zero tolerance toward bullying or increased surveillance to monitor and address bullying behavior.	*“Have firm anti bullying policies that are enforced.”*	172 (30.8%)
Nothing/unattainable goal	This code captures responses that express the belief that nothing can be done to stop weight‐related bullying in schools. It includes perspectives suggesting the issue is beyond the school's control and requires broader societal change, as well as responses that recommend not addressing the issue at all, often out of concern that bringing attention to it may exacerbate the problem.	*“I don't think that there is much a school can do to stop this kind of behavior”*	92 (16.5%)
Discontinuation of harmful practices	This code refers to the recommendation to stop practices that may contribute to body image issues, weight stigma, or bullying. It includes responses advocating for the discontinuation of weight‐focused activities, such as fitness testing, weighing students in class settings, or promoting weight loss, which may reinforce harmful stereotypes or trigger negative feelings about body image.	*“Stop putting such emphasis on weight loss in health class”*	41 (7.3%)
Support services	This code refers to providing assistance and resources to targets of bullying. It includes responses that emphasize the importance of listening to and supporting the bullying target, taking their experiences seriously, offering anonymous reporting options, and providing access to resources such as professional referrals or counselors.	*“The biggest thing in my opinion is to have a support system for students who experience it, so they can talk to an authority figure who can help sort out the issue, whether that be confronting the bully or addressing classes/the school at large”*	36 (6.4%)
Victim blaming	This code refers to responses that place responsibility for weight‐related bullying on the target of the bullying, suggesting that those who are bullied “deserve it” or are at fault. It includes responses that propose weight loss as a solution to bullying, implying that the target of the bullying should change to avoid being targeted.	*“Make kids not fat”*	29 (5.2%)
Role modeling	This code refers to the importance of teachers and other role models demonstrating positive behaviors to influence students. It includes responses suggesting that schools should set an example by modeling kindness, respect, inclusivity, and empathy, encouraging students to adopt these behaviors in their interactions with others.	*“Teachers and schools should set an example and model good behavior”*	25 (4.5%)
Uncategorized	This code includes responses where participants express uncertainty or provide answers that do not clearly align with any of the codes.	*“I'm honestly not sure”*	65 (11.6%)

^a^ Codes generated from a content analysis of responses to one question (Q4: What do you think schools can do to stop weight‐related bullying and teasing?). Responses may receive multiple codes, so column totals are > 100%.

### 2.3 Support for Anti‐Bullying Policies Including Weight‐Related Bullying

Support for anti‐bullying policies that specifically address weight‐related bullying and teasing was assessed through one question (Q5: “Do you think anti‐bullying policies at schools should specifically include weight‐related bullying and teasing? Why or why not?”; see Table 4 for code descriptions, illustrative quotes, and frequencies). The majority of respondents (68.5%) supported policies that explicitly included weight‐related bullying. Among those who provided rationale for their affirmative response, the two most common reasons were the importance of raising awareness and addressing weight‐related bullying (32.0% of affirmative responses; e.g., “Yes because it is a huge issue that effects so many children especially young girls.”) and the need to include all types of bullying in anti‐bullying policies to ensure no vulnerable student identities are neglected (31.0% of affirmative responses; e.g., “I think it should cover everything because all forms of bullying is bad”). A smaller proportion (2.9% of affirmative responses) emphasized that weight should be included to acknowledge the lack of control individuals may have over their weight (e.g., “It's something that some people can't change.”).

**TABLE 4:** Support for weight‐specific anti‐bullying policies^a^ response codes.

Code Title	Description	Illustrative quote	Frequency, *N* (%) (*n* = 550)
Affirmative; support for including weight‐related bullying in anti‐bullying policies	This code refers to responses that directly affirm the need for schools to specifically include weight‐related bullying and teasing in their anti‐bullying policies. This includes explicit mentions of support or agreement, such as the use of the word “yes” or other affirmative phrases indicating advocacy for such inclusion.	*“Yes because it is something that can hurt the victims”*	377 (68.5%)

Affirmative subcodes			*N* (% of all responses, % of affirmative responses)
Raise awareness; importance of addressing weight‐related bullying	This subcode captures responses highlighting the benefits of anti‐bullying policies in raising awareness, increasing knowledge, or making a positive difference (e.g., preventing harm, improving mental health). This code also includes responses that stress the significance or prevalence of weight‐related bullying.	*“Yes. It's a common reason people get teased and it leads to poor mental health outcomes”*	176 (32.0%, 46.7%)
Inclusion of all types of bullying in anti‐bullying policies (affirmative)	This subcode captures responses that advocate for the inclusion of all forms of bullying (including but not limited to weight‐related bullying) in anti‐bullying policies. These responses emphasize the importance of addressing every type of bullying, without exclusion, in school policies.	*“Yes, it's a form of bullying, people get hurt because of this”*	117 (21.3%, 31.0%)
Weight controllability	This subcode captures responses that emphasize the idea that individuals may not have control over their weight or are not at fault for their weight. This includes perspectives acknowledging that weight‐related bullying or teasing is unfair because weight can be influenced by factors beyond an individual's control, such as genetics, health conditions, or other external factors.	*“Yes it should, some students can't help the issues of their weight and don't deserve to be bullied because of that”*	11 (2.0%, 2.9%)
Opposing; opposition to including weight‐related bullying in anti‐bullying policies	This code captures responses that directly express opposition to the inclusion of weight‐related bullying and teasing in anti‐bullying policies. This includes explicit mentions of disagreement or refusal, such as the use of the word “no” or other phrases that indicate a lack of support for including weight‐related bullying in such policies.	*“No”*	150 (27.3%)

Opposing subcodes			N (% of all responses, % of opposing responses**)**
Inclusion of all types of bullying in anti‐bullying policies (opposing)	This code captures responses that argue there is no need to make a distinction between different types of bullying, including weight‐related bullying. These responses advocate for treating all forms of bullying equally in anti‐bullying policies, without singling out specific types such as weight‐related bullying.	*“No. I think any bullying should be disciplined regardless of the reason.”*	66 (12.0%, 44.0%)
Unintended consequences; Unwanted attention	This code captures responses that highlight concerns about the potential negative effects of implementing weight‐specific anti‐bullying policies. This includes fears that such policies might draw excessive attention to weight issues, potentially singling out individuals or groups in a harmful way. It also includes concerns about unintended consequences or problems that may arise from focusing on weight as a specific category in anti‐bullying efforts.	*“No. Students are more likely to bully or tease others about something, or even make fun of that thing in general or behind another student's back, if they are explicitly told not to do it.”*	57 (10.4%, 38.0%)
Ineffective policies	This code captures responses that express skepticism or criticism about the effectiveness of anti‐bullying policies, particularly in reducing bullying. This includes perspectives that argue such policies will not lead to meaningful change or will not effectively address or prevent bullying behaviors, including weight‐related bullying.	*“a lot of the policies don't work anyways so I dont see why it would help”*	31 (5.6%, 20.7%)
Unhealthy lifestyle concerns	This code captures responses that suggest being “overweight” is inherently unhealthy or raise concerns that weight‐specific anti‐bullying policies might inadvertently encourage or condone unhealthy behaviors, lifestyles, or weight. This includes perspectives that link weight with health outcomes and caution against normalizing or accepting higher weight as acceptable without addressing potential health risks.	*“No, being overweight is unhealthy”*	9 (1.6%, 6.0%)
Uncategorized	This code includes responses where participants express uncertainty (“Don't know”) or provide answers that do not clearly align with any of the codes.	*“i don't know if it's necessary, i've never seen someone being bullied/teased because of their weight”*	23 (4.2%)

^a^ Codes generated from a content analysis of responses to one question (Q5: Do you think anti‐bullying policies at schools should specifically include weight‐related bullying and teasing? Why or why not?). Responses may receive multiple codes, so column totals are > 100%.

Among the 27.3% of respondents who opposed the explicit inclusion of weight‐related bullying in anti‐bullying policies, many (44.0% of opposing responses) argued that all types of bullying should be addressed equally within anti‐bullying policies, without the need to differentiate between them (e.g., “no that's dumb lol. bullying is bullying and if you specifically target one type of bullying then another will just pop up.”). Others raised concerns about unintended consequences of such policies (38.0% of opposing responses), such as the potential for encouraging undue focus on appearance (e.g., “I fear that instituting these types of policies will only place a target on the backs of these individuals”). 20.7% of opposing responses questioned the effectiveness of such policies in reducing bullying (e.g., “I just don't think bullying is the problem lol. Like having a policy focused on weight‐related bullying is a bandaid on a bullet hole. Why are kids bullying fat kids? Because they're taught at home and on TV that it's bad to be fat. So you can have policies to target weight related bullying, but the real solution is to teach people to not be fatphobic.”), while a smaller proportion (6.0%) cited rationale focused on an individual's responsibility to control their weight (e.g., “No because people need to get in shape.”).

## 3 Discussion

The results of this study provide valuable insights into youth perspectives on weight‐related bullying and potential approaches schools can take to address this significant public health issue. The most commonly identified school‐based anti‐bullying strategies were education to raise awareness and programs focused on weight‐based bullying. However, more than one‐quarter of respondents also suggested policy solutions, such as implementing consequences for bullying. A majority of respondents supported explicitly including weight in school anti‐bullying policies. Those who opposed its inclusion argued that all forms of bullying should be treated equally, expressed concerns about unintended consequences, and questioned the effectiveness of such policies.

Our findings illustrate the pervasiveness of weight‐based bullying in school settings. A significant proportion of youth in our sample (70.6%) had witnessed weight‐related bullying, and they identified schools as a primary setting for in‐person bullying. This finding aligns with a previous study, which found that 78.5% of high school students reported observing weight‐related bullying either sometimes, often, or very often [20]. While characteristics of both the individual doing the bullying and characteristics of the target of the bullying were frequently cited as contributing factors in bullying, target‐related qualities were more commonly cited as explanations for weight‐related bullying (46.5% of responses) compared to general bullying (28.7% of responses). This focus on target‐related factors highlights the unique complexities of addressing bullying based on weight and reflects a tendency toward victim‐blaming. At times, this language was overt, while in other instances, victim‐blaming was subtly expressed within seemingly caring responses, a phenomenon often referred to as “concern trolling” [21].

Additionally, these target‐focused responses could represent attitudes that reflect commonly held beliefs that a person's weight is a result of personal choices, rather than the result of a complex interplay of social, environmental, and genetic factors. The extent to which a bullied individual is perceived to have “control” over their body size may demonstrate an implicit bias, where blame is attributed to the target of the bullying [22]. This aligns with the perspectives shared by a small number of participants in our study, whose remarks explicitly held the target of the bullying at fault. Furthermore, public health messaging related to weight frequently emphasizes personal responsibility over community level and systemic supports, potentially reinforcing harmful stigma [1, 2, 7, 8, 23, 24, 25]. Importantly, there is a broad consensus among weight stigma experts that weight loss should not be proposed as a response to weight stigma [26].

Youth perspectives in this study reinforce the idea that schools are not only sites where weight‐based bullying occurs but also environments that may perpetuate stigma. Some respondents (7.3%) highlighted ways in which schools may unintentionally reinforce weight‐stigmatizing attitudes, which can then be projected onto youth. These participants pointed to practices such as fitness testing, BMI screenings, and weighing students in public settings, which may encourage comparisons and reinforce a focus on weight. Practices like BMI report cards, which notify parents of their child's weight status, have been criticized as ineffective and potentially harmful, with some studies linking them to increased disordered eating and worsened mental health outcomes [27, 28]. Despite intentions to promote health, weight‐focused programs may inadvertently legitimize weight‐based bullying by framing higher body weight as inherently negative or undesirable, thereby contributing to bullying and exclusion [29]. Additionally, educators themselves may play an unintentional role in maintaining a weight‐stigmatizing environment. Research shows that weight bias in educational settings negatively impacts students' well‐being and academic experiences [30], and supports recommendations that caution against weight‐focused practices, advocating instead for policies and practices that promote inclusivity, body positivity, and acceptance to promote holistic health, while minimizing unintended negative consequences [25, 31, 32].

The majority of participants favored explicitly including weight in school anti‐bullying policies. However, a significant minority (27.3%) opposed this inclusion. Some participants expressed doubts about the effectiveness of these policies, reflecting a sense of helplessness about addressing weight‐based bullying, perhaps due to the pervasive influence of weight stigma and diet culture in society. These concerns underscore the importance of pursuing environmental and policy‐level interventions where possible. School‐based policies represent a promising avenue for shifting societal narratives about weight. Just as ideas about weight from society filter into schools, schools can shape student attitudes and behaviors and, over time, contribute to broader cultural change. Among those who opposed enumeration of weight in school‐based policies, some emphasized the importance of treating all identities equally without prioritizing any group, raised concerns about potential unintended consequences, or endorsed the misconception that weight‐related bullying could somehow benefit the health of the target of the bullying. While these views were in the minority, they mirror concerns raised by some lawmakers and constituents in states considering weight‐related bullying legislation. For example, when Colorado and other states debated including weight‐related bullying protections, testimony from youth and family members impacted by weight‐related bullying was met with concerns about potential harms to students with more idealized body types and ideological debates about whether certain identities deserve specific legal protection. Youth in our study who opposed enumeration similarly expressed concerns that highlighting one identity may detract from others or cause division. While these concerns are important to acknowledge, evidence suggests that enumerated policies are more effective in reducing bullying and supporting targeted students [33]. Advocates must continue to communicate the value of enumeration not as a means of prioritizing one group over another, but as a strategy for ensuring that commonly targeted groups receive the specific protections they need.

To build public and legislative support for weight‐related bullying protections, advocates should engage youth and stakeholders in advocacy, educate lawmakers and the public through targeted campaigns, and involve those affected in the process to address concerns about fairness and effectiveness [34]. Helping youth, their parents, and legislators recognize the systemic benefits of specific anti‐bullying policies, in addition to the barriers to enforcement (and human cost) when specific protections are not in place, may aid lawmakers in bolstering more effective policy solutions. State‐level anti‐bullying policies that explicitly include weight can reduce controversy by removing the burden from individual school districts to draft, debate, and defend their own language. These policies set a clear precedent, promote consistency across districts, and draw attention to implementation. However, without proper training, oversight, and leadership commitment, even well‐written policies may fall short. Future efforts must focus not only on policy language but also on ensuring schools are prepared and supported to implement these protections effectively and equitably.

### 3.1 Implications for School Health Policy, Practice, and Equity

Although some oppose policies targeting weight‐related bullying, previous research on anti‐bullying laws demonstrates their potential to improve youth outcomes. While no studies have specifically examined the impact of anti‐bullying policies targeting weight stigma on youth, a study by Rees and colleagues found that state‐level anti‐bullying laws led to reductions in bullying, depression, and suicide rates among adolescents [9]. This study, which used data from 21 states, found that female and LGBTQ+ youth experienced the largest improvements. Additionally, the analysis supported a causal interpretation, suggesting that the policies directly contributed to these positive health measures. These findings suggest that state‐level anti‐bullying laws, particularly those with stronger provisions such as clear definitions, required enumerations of protected characteristics (i.e., weight), mandated reporting procedures, and enforcement mechanisms, could also help address weight‐related bullying and its associated health impacts.

However, policy alone is not sufficient. An important implication for school health is the need to examine the broader school environment for practices that may inadvertently reinforce weight bias. This includes reviewing what is taught about weight in health and physical education classes, how food and body size are discussed, and how wellness policies frame health. Educational interventions should address common myths about weight and health, particularly the belief expressed by some youth in this study that weight‐based bullying may be helpful or motivating. Schools should also evaluate whether their environments are physically and socially inclusive for students in larger bodies. In addition, schools should support training for educators and other staff on topics such as weight stigma, eating disorders, and inclusive health messaging to ensure that all school personnel are equipped to foster a supportive and respectful environment for all students.

To ensure the success of weight‐related bullying policies, it is essential that they are well‐designed, evidence‐based, and forward‐thinking, as ineffective policies could erode public trust, undermining their effectiveness [34]. Iyer recommends several strategies to mitigate objections to diversity, equity, and inclusion policies, such as linking policies to principles of justice, emphasizing broader group interests, and focusing on the positive aspects of disadvantaged groups [34]. While these strategies have shown promise, further research is needed to determine best practices. Importantly, the youth voice must be central to the development and implementation of school‐based anti‐bullying laws and policies. As this study demonstrates, young people offer critical insights into both the causes of and potential solutions to weight‐based bullying. Schools, districts, and policymakers should actively involve students, particularly those whose body types deviate from idealized standards and those with lived experience of bullying in shaping and evaluating anti‐bullying strategies. Doing so ensures policies reflect the realities of students' lives and helps shift the broader cultural narrative around body size and bullying. As legislation targeting weight‐related discrimination expands, it will be critical to collect timely research on the impact of these policies to ensure their effectiveness and minimize unintended consequences.

### 3.2 Limitations

While this study provides valuable insights into youth perspectives on policy‐focused interventions for weight‐related bullying, several limitations must be acknowledged. First, the study did not collect data on participants' body size, weight perception, or internalized weight bias, which could have provided additional context for interpreting their perspectives. Additionally, previous research suggests that youth‐reported preferences for bullying interventions may not align with the effectiveness of these programs, underscoring the importance of involving youth throughout the research and evaluation process to ensure strategies for preventing weight‐related bullying are both effective and relevant to their needs [15, 35]. Although the wide age range of participants may be viewed as a limitation due to differences in developmental perspective, it also serves as a strength by capturing a broader spectrum of experiences. Younger participants may not yet have encountered bullying in high school settings, while older participants who are no longer in high school can offer insights shaped by a wider range of school experiences. Finally, this study focused solely on youth perspectives and did not include input from educators or other school personnel, who are critical stakeholders in the implementation of anti‐bullying policies.

## 4 Conclusions

The findings from this study shed light on the complexities of weight‐related bullying from the perspectives of youth, revealing both the pervasive nature of this issue and the potential for policy‐driven solutions. While many youth emphasized the importance of education and programming in addressing weight‐related bullying, a substantial number also advocated for formal policies that include consequences for weight‐related discrimination within anti‐bullying frameworks. These results suggest that incorporating weight explicitly into school policies could provide a more supportive environment for students affected by weight stigma. The study also highlights the need for careful consideration of both the direct and indirect ways schools and society perpetuate weight‐related stigma, underscoring the importance of adopting inclusive, body‐positive approaches in educational settings [29]. As such, this research contributes to the growing body of evidence that supports comprehensive, research‐backed policies that can better protect youth from weight‐related bullying and its harmful effects on mental health. Further investigation into the impact of such policies is essential for refining effective strategies to address this ongoing public health challenge.

## Conflicts of Interest

The authors declare no conflicts of interest.

## Data Availability

The data that support the findings of this study are available from the corresponding author upon reasonable request.
